# Ventricular fibrillation by anaphylaxis following consumption of blue_skinned fish

**DOI:** 10.1002/ams2.144

**Published:** 2015-08-12

**Authors:** Saori Otsubo, Takashi Nishiyama, Naoki Okada, Yukihiro Ando, Katsumi Yamada, Yuji Maeda, Yusuke Nishimura

**Affiliations:** ^1^ Departments of Disaster and Emergency Medicine Kobe University Hospital Kobe Hyogo Japan

**Keywords:** Anisakis allergy, cardiopulmonary arrest, Kounis syndrome, shock

## Abstract

**Case:**

Approximately 4_h after eating mackerel sushi, a 65_year_old man developed generalized itchiness and redness, after which he became unresponsive. He went into a state of ventricular fibrillation but after he was defibrillated twice, his heartbeat returned. The electrocardiogram obtained immediately after hospitalization showed some ST_segment depression, but a subsequent electrocardiogram showed improvement. Coronary CT showed no obvious stenosis or plaque in the coronary artery.

**Outcome:**

Results of an IgE_RAST test confirmed that the level of allergen_specific IgE for mackerel measured <0.10_UA/mL, while the level for anisakis was 1.91_UA/mL.

**Conclusions:**

As for the mechanism leading to cardiac arrest, it is thought that the histamines and leukotrienes released from cardiac mast cells caused a coronary artery spasm (Kounis syndrome). This anaphylactic shock is considered to be a result of anisakis allergy, but in general cases of anaphylaxis resulting from consumption of blue_skinned fish.

## Introduction

We report a case of ventricular fibrillation caused by anaphylactic shock after the intake of blue_skinned fish and also discuss the related developmental mechanism and diagnosis for allergy.

## Case

**Figure 1:**
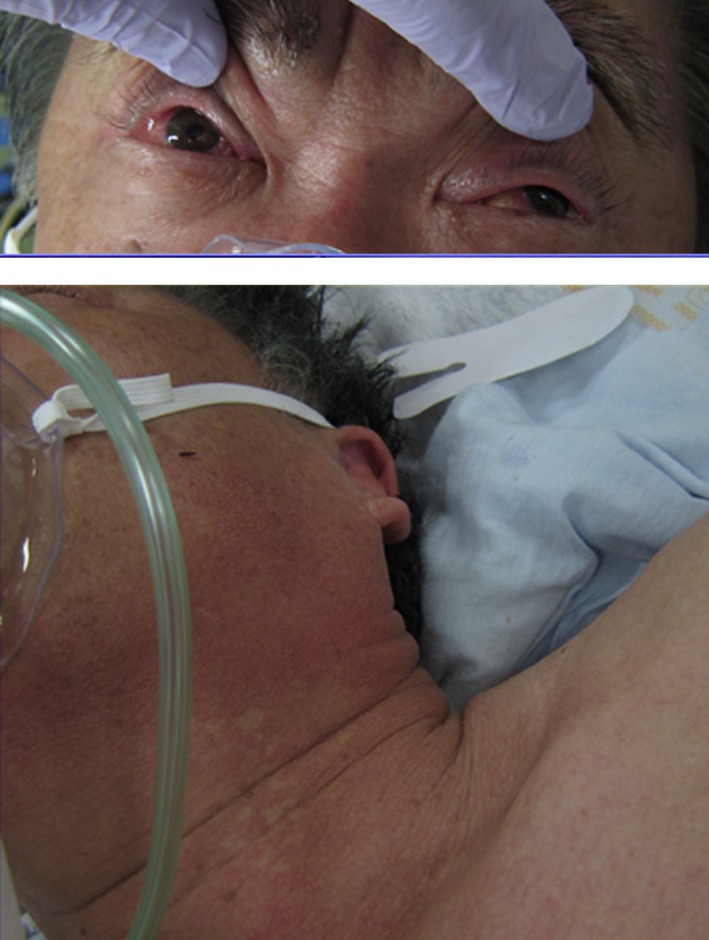
The patient was observed to have hyperaemia of the bulbar conjunctiva and flushing of the face on arrival.

**Figure 2:**
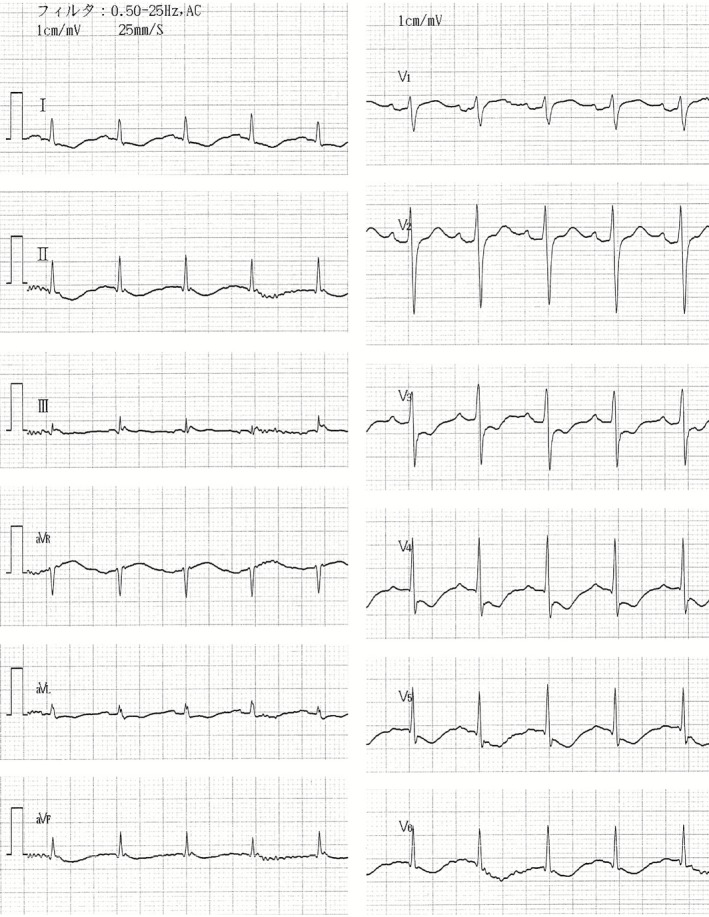
Electrocardiogram finding immediately after arrival. A 12_lead electrocardiogram showed that ST had dropped to a lower level with I, aVL, and V3_6.

After a 65_year_old man had mackerel sushi for dinner, he experienced itching, flushing, and a sense of overall exhaustion approximately 4_h later, followed by difficulty in breathing and lack of response to verbal cues. According to his medical history, he had been taking amlodipine besylate and candesartan cilexetil prescribed by a local doctor for high blood pressure. He had also experienced skin flare_ups, itching and vomiting as a result of blue_skinned fish consumption since he was in his 40s, but because those symptoms did not occur each time, he had continued eating the fish occasionally without such symptoms during the previous 10 years. His family medical history showed that his father died of stomach cancer. When the emergency medical assistance services arrived at his home, the patients remained conscious, but palpation of the carotid artery showed slight weakness, blood pressure was immeasurable and marked flushing over his entire body was noted. On the way to the hospital, spontaneous but open_mouthed breathing became weaker, so that assisted ventilation was started with a bag valve mask. Because ventricular fibrillation (VF) was noted in the ambulance, chest compression was initiated and electric shock was administered with an automated external defibrillator (AED). However, when assessment after 2_min showed that VF was continuing, electric shock by AED was administered again. Return of spontaneous circulation was confirmed when the patient arrived at the hospital (cardiac arrest had lasted approximately 5_min). On admission, he was unconsciousness with the following vital data: GCS_=_E1V2M5 (no pupillary abnormalities); respiratory rate 40_r.p.m. (clear sound, no stenosis was heard); heart rate 115_b.p.m. (irregular rhythm); blood pressure 112/83_mmHg; SpO2 98% (with administration of 10_L/min by means of a mask); axillary temperature 35.5_C. No obvious injury was detected on the body surface, but flushing of the head and neck area and redness of the bulbar conjunctiva were noted (Fig._1). A 12_lead electrocardiogram obtained immediately after arrival showed that ST had dropped to a lower level with I, aVL, and V3_6 all recognizable (Fig._2). On the basis of cardiac ultrasonography findings, it was decided that an emergency cardiac catheter test need not be used because of favorable wall motion and EF of 74%. The complete blood count showed no increase in eosinophils or IgE. Diagnosis of anaphylactic shock based on medical interview and physiological findings led to massive administration of acetated Ringer's solution and massive administration of hydroxyzine, methylprednisolone, and famotidine. Level of consciousness in the intensive care unit improved to GCS_=_E4V5M6, and respiratory condition and circulatory dynamics also became stabilized. The peak levels of creatine kinase and creatine kinase_MB after 6_h from the time of admission were found to be 365_U/L and 23_U/L, respectively, and a 12_lead electrocardiogram demonstrated that the ST level had also improved. Coronary CT conducted on the 8^th^ day indicated hypoplasia in the left coronary artery, and especially in the left anterior descending artery, but marked stenosis and plaque were not recognized in the coronary artery. Results of the radioallergosorbent test (IgE_RAST) using an IgE capsulated hydrophilic carrier polymer were <0.10_UA/mL for mackerel and 1.91_UA/mL for anisakis (less than the reference value of 0.34_UA/mL). The patient was discharged from hospital on the 12^th^ day showing no neurological sequelae.

## Discussion

It is thought that the causative mechanism of cardiac arrest by anaphylaxis is hypoxia or myocardial ischemia resulting from airway obstruction due to edema of the larynx.^1^ Because no airway stenosis was recognized in the case presented here, we determined that VF was caused by myocardial ischemia.^2^ As for the causative mechanism of cardiac arrest produced by anaphylaxis, it was previously assumed that myocardial ischemia could occur as a result of a reduction in circulating blood volume and diminishing of the coronary blood flow. However, at present the main consensus is that coronary artery spasm can result from anaphylaxis caused by histamine or leukotriene released from cardiac mast cells as the target cells. Such a coronary artery spasm accompanied by anaphylaxis is also known as Kounis syndrome.^3^ There are two reports describing a patient with coronary artery spasm caused by Kounis syndrome suffering cardiac infarction or a full recovery of cardiac function without myocardial necrosis.^4^, ^5^, ^6^ A myocardial prognostic factor would thus be duration of the coronary artery spasm and level blood pressure as well as whether cardiovascular risk for and arteriosclerotic lesion of the coronary artery would be involved.^7^ In the case presented here, diminished ST in the region of the left ventricular wall (I, aVL, V3_6) was observed on a 12_lead electrocardiogram at the time of the patient's arrival at the hospital, so it was thought that coronary artery spasm had occurred in conjunction with Kounis syndrome. The reason why the patient recovered from this condition without development of cardiac infarction in spite of hypoplasia of the left anterior descending coronary artery would be the continual performance of cardiopulmonary resuscitation, with bystander CPR as one of the factors, until return of spontaneous circulation. Anisakis simplex is a nematode of Ascaridida: Anisakidae and many third_stage larvae can become parasitic in the abdominal cavity of marine fishes and Pacific flying squid. It has been maintained for a long time that an allergy can occur as a result of consumption of fish as evidenced by the use of such terms as fish allergy or blue_skinned fish allergy. It was once considered that antigenicity in fish protein could cause such allergic symptoms, but according to some reports,^8^ anisakis simplex, which is parasitic in fish, might be the cause. We suspected anisakis allergy in our case, although the patient mentioned no symptoms following consumption of blue_skinned fish during the medical interview. As mentioned previously, anisakis simplex is parasitic in many marine fishes, not only mackerel but also cherry salmon and Pacific flying squid. Furthermore, the antigen for anisakis simplex cannot be deactivated completely even by heating to 100_C for approximately 15_min,^9^ and, according to some reports, allergic symptoms can even develop after intake of boiled fish. The widespread dissemination of information about the need for caution regarding consumption of marine fish in addition to mackerel is therefore of vital importance, including warnings that allergic reaction may occur even with heat_treated marine fish and familiarization with the food allergy known as anisakis allergy.

## Conclusion

We examined a case with VF caused by anaphylactic shock after intake of blue_skinned fish. It is thought that the causative mechanism of this state was histamine or leukotriene released from cardiac mast cells as target cells after development of anaphylaxis with anisakis as the antigen. This shows the need for widespread dissemination of information that a form of anaphylaxis after eating blue_skinned fish can be caused by anisakis as the antigen.

## Conflict of Interest

None.
